# Supplementary material for: A set of multi-entry identification keys to African frugivorous flies (Diptera, Tephritidae)
Source: Zookeys. 2014 Jul 24;(428):97–108. doi: 10.3897/zookeys.428.7366 (PMC4143993; doi:10.3897/zookeys.428.7366)
Supplement: Supplementary material 6 — Key to Ceratitis [file zookeys-428-097-s006.zip › SF6_ZooKeys_key to Ceratitis/key/SF6_ZooKeys_key to Ceratitis/Media/Html/desc_Ceratitis_copelandi.html]

Natural Language Description


# A set of multi-entry identification keys to African frugivorous flies (Diptera, Tephritidae)

### Massimiliano Virgilio, Ian White, Marc De Meyer

## Ceratitis copelandi

(key to Ceratitis) abdomen (key to Ceratitis) 80. abdomen spot pattern unspotted, (key to Ceratitis) 81. tergite 2 with silvery grey band yes, (key to Ceratitis) 82. tergite 4 with silvery grey band yes, (key to Ceratitis) 83. tergite 3 with brown-black band or completely black yes, (key to Ceratitis) 84. (females) oviscape length shorter than abdominal tergites, at most as long, (key to Ceratitis) 85. (females) aculeus tip simple, without notch, identations, serrations or appendages no, (key to Ceratitis) 86. (females) aculeus tip with small notch only and no indentations, serrations or appendages yes, (key to Ceratitis) 87. (females) aculeus pointed, tip with subapical step only and no indentations, serrations or appendages no, (key to Ceratitis) 88. (females) aculeus tip uniformly serrated no, modified in different way, (key to Ceratitis) 89. (females) aculeus tip serrated and with lateral protuberances above the serrations no, modified in different way, (key to Ceratitis) 90. (females) aculeus tip serrated with large blad-like serrations no, (key to Ceratitis) 91. (females) aculeus tip with large indentations, broadening upwards modified in different way, (key to Ceratitis) 92. (females) aculeus distal end elongated but straight no, not as such. (key to Ceratitis) head (key to Ceratitis) 10. number of frontal setae three or two, (key to Ceratitis) 11. upper frontal setae well developed no (poorly developed) or yes, (key to Ceratitis) 13. (males) superior orbital setae incrassate no, normal, (key to Ceratitis) 14. (males) lower orbital setae modified, flattened to some degree no, (key to Ceratitis) 19. face, ground colour white to yellow, (key to Ceratitis) 2. first flagellomere brown to black or yellow to red, (key to Ceratitis) 20. face with transverse median band no, (key to Ceratitis) 21. face with upper half darker coloured no, (key to Ceratitis) 22. lower margin of face same colour as rest of face yes, (key to Ceratitis) 23. lateral vertical setae black yes, (key to Ceratitis) 24. mouthparts no, pale, at most isolated dark patches in upper part, (key to Ceratitis) 25. occiput in upper part dark no, (key to Ceratitis) 26. head with 2 orbital setae yes, (key to Ceratitis) 27. frons with medilongitudinal shining line yes, (key to Ceratitis) 3. arista hairing short hairs, (key to Ceratitis) 4. (males) frons enlarged and inflated no, normal in shape, (key to Ceratitis) 5. frons longer than wide yes, (key to Ceratitis) 6. frons with silvery shine, at least in some areas yes, (key to Ceratitis) 7. (males) ocellar tubercle on protuberance no or yes, (key to Ceratitis) 8. ocellar setae colour black, (key to Ceratitis) 9. frontal setae yes. (key to Ceratitis) legs (key to Ceratitis) 63. fore coxa black no, (key to Ceratitis) 64. fore coxa with silvery patch no, (key to Ceratitis) 66. (males) fore femur with anteroventral dense row of silvery hairs no, (key to Ceratitis) 67. (males) fore femur posteriorly with bush of hairs no or yes, (key to Ceratitis) 68. (males) fore femur posterior bush colour black or pale, (key to Ceratitis) 69. (males) mid femur, ventrally with feathering (even only partly) no, (key to Ceratitis) 73. (males) mid tibia with feathering yes, (key to Ceratitis) 74. (males) mid tibia feathering extension in apical part reaching less than half-way, (key to Ceratitis) 75. (males) feathering on mid tibia - coloured dark, (key to Ceratitis) 76. (males) mid tibia flattened and broadened no, (key to Ceratitis) 77. (males) mid tibia anterior side with long (and dispersed) hairs no, (key to Ceratitis) 78. (males) mid tibia anterior side with silvery shine when viewed from certain angle no, (key to Ceratitis) 79. (females) fore femur with dark hairs between posterior and posterodorsal row of bristles no. (key to Ceratitis) sex female or male. (key to Ceratitis) thorax (key to Ceratitis) 28. postpronotal lobe with distinct brown or black spot no, at most darker yellow, (key to Ceratitis) 30. scutum dark streaks or spots unclear streaks and darker markings; markings usually obvious when viewed from behind, obscured when viewed from front, (key to Ceratitis) 32. (male) scutum with anterior third darker, sickle-shaped dark coloration no, (key to Ceratitis) 33. postsutural (pale or yellow) lateral lines present no, (key to Ceratitis) 34. prescutellar markings present yes, (key to Ceratitis) 36. scutellum colour form marked, (key to Ceratitis) 37. scutellum markings trilobed (joint or separate), (key to Ceratitis) 39. base scutellum with 2 brown or black spots no, at most localized darker coloring, (key to Ceratitis) 41. spots at end scutellum separate, (key to Ceratitis) 43. apical spots extent only reaching till halfway of scutellum at most or reaching till basal third or fourth of scutellum, (key to Ceratitis) 44. anepisternal setae one, (key to Ceratitis) 45. anepisternum pilosity whole pale pilosity, except few darkish hairs along upper margin, (key to Ceratitis) 46. anepisternum colour lower half darker coloured, (key to Ceratitis) 47. lower part anepisternum brown to black or darker yellow to orange. (key to Ceratitis) wings (key to Ceratitis) 53. wing with anterior apical band forming continuous band with anterior part of discal band (at most connection partly interrupted) no, (key to Ceratitis) 54. discal band width narrower, not joined with basal spots, (key to Ceratitis) 55. subapical band free, (key to Ceratitis) 56. posterior apical band absent, or poorly developed or free, (key to Ceratitis) 57. wing with longitudinal band along vein M no, (key to Ceratitis) 58. wing banding reduced to isolated spot(s) no, (key to Ceratitis) 59. crossvein r-m placed before middle of cell dm, (key to Ceratitis) 60. (males) wing with subapical incision lobe no, (key to Ceratitis) 61. (males) wing apical tip shape rounded.
